# Concomitant Hypodontia and Hyperdontia (CHH): Clinical Challenges and Public Health Strategic Importance in Saudi Arabia

**DOI:** 10.7759/cureus.117642

**Published:** 2026-10-08

**Authors:** Alhassan Alshehri, Turki Alahmari, Waad Aldaghriry, Razan Aljarboua, Khalid Alshalhoub

**Affiliations:** 1 Pediatric Dentistry, North of Riyadh Dental Center, Riyadh Second Health Cluster, Riyadh, SAU

**Keywords:** concomitant hypo-hyperdontia, hypodontia, kingdom of saudi arabia (ksa), pediatric dentistry, public health epidemiology health systems, public health problem, public health system, saudi arabia 2030 vision

## Abstract

Concomitant hypodontia and hyperdontia (CHH), or "hypo-hyperdontia", represents a rare numerical dental anomaly in which a patient simultaneously presents with congenitally missing teeth and supernumerary elements. This narrative review examines the genetic and molecular mechanisms underlying CHH, focusing on the dual disruption hypothesis of dental lamina development, alongside global epidemiological data and reported male predominance. Clinical presentation, radiographic diagnosis, and multidisciplinary management strategies - encompassing surgical, orthodontic, and restorative interventions - are critically evaluated. A structured clinical decision-making framework and age-specific management pathways are proposed to guide practitioners. The psychosocial implications of CHH, particularly in adolescents, are discussed. The Saudi Arabian healthcare context is briefly addressed as a case example of integrating rare dental anomaly management into a transforming primary care system. This review acknowledges its narrative design and the limitations of the current evidence base and highlights critical gaps requiring future epidemiological and clinical research.

## Introduction and background

The orchestration of human odontogenesis is a highly regulated biological process governed by intricate molecular signaling networks and epithelial-mesenchymal interactions. Despite this precise developmental control, the dentition remains susceptible to various developmental disturbances that manifest as numeric anomalies. Traditionally, these anomalies are bifurcated into two mutually exclusive categories: hypodontia, defined as the congenital absence of one to five teeth excluding third molars, and hyperdontia, the presence of teeth more than the normal dental formula [1]. While both conditions are frequently encountered in isolation within general dental practice, their simultaneous occurrence in a single, non-syndromic individual is a rare clinical phenomenon known as concomitant hypodontia and hyperdontia (CHH) or "hypo-hyperdontia" [2,3].

First formally characterized in the dental literature in the mid-twentieth century, CHH represents a unique "numerical paradox" where the dental lamina appears to exhibit simultaneous hyperactivity and localized failure [2,4]. Within the anterior maxilla, often referred to as the "esthetic zone", this anomaly frequently involves the maxillary lateral incisor. The lateral incisor is the second most common tooth to be congenitally missing (agenesis), with a reported prevalence between 1.91% and 3.6% in various populations [3,5]. When this agenesis is complicated by the presence of a supernumerary tooth, such as a mesiodens or a tuberculate duplicate, it creates a significant "space crisis" that disrupts the eruption path of adjacent permanent teeth and compromises occlusal stability [5,6].

Global epidemiological data indicate that CHH is rare, with published prevalence estimates ranging from 0.002% to 3.1% across different study populations [4,7]. This wide range reflects heterogeneity in study design, population selection, and diagnostic criteria - specialist referral cohorts consistently yield higher estimates than population-based radiographic screening studies - rather than true biological variation. The condition demonstrates a consistent male predominance, with reported male-to-female ratios ranging from approximately 1.5:1 to 2.4:1 [1,8].

This narrative review synthesizes current knowledge on CHH, covering its molecular basis, global epidemiology, clinical diagnosis, and multidisciplinary management. A structured clinical decision-making framework is proposed to support General Dental Practitioners (GDPs) and specialists in managing this rare condition. The Saudi Arabian healthcare context is referenced briefly as a relevant example of primary care integration, without overstating CHH's broader public health significance.

## Review

Methods

This narrative review was conducted in accordance with the Scale for the Assessment of Narrative Review Articles (SANRA) framework. A structured search of PubMed/MEDLINE, Google Scholar, Scopus, and Web of Science was performed in January-February 2026 using the following search strings: (“concomitant hypodontia hyperdontia”) OR (“hypo-hyperdontia”) OR (“simultaneous tooth agenesis AND supernumerary”) OR (“CHH”), combined individually with terms including “prevalence,” “genetics,” “pathogenesis,” “management,” “diagnosis,” and “Saudi Arabia.” No lower date restriction was applied in order to capture seminal historical reports, including those predating 2000; the upper boundary was February 2026. Approximately 200 records were retrieved across databases. After deduplication and title/abstract screening for relevance to CHH, approximately 60 full-text articles were reviewed, of which 18 were selected for inclusion in this review. Inclusion criteria encompassed case reports, case series, cross-sectional studies, cohort studies, and prior reviews addressing CHH or directly informing its pathophysiology, epidemiology, or management. Studies on isolated hypodontia or isolated hyperdontia were included only when directly relevant to understanding CHH mechanisms or treatment principles. A formal meta-analysis was not performed because no CHH-specific pooled analyses exist and the available studies are too heterogeneous in design, population, and diagnostic criteria to permit valid quantitative synthesis. This narrative approach does not include formal quality assessment or risk-of-bias evaluation; the evidence synthesis is therefore interpretive, and findings should be read in the context of the limitations described in the Limitations section.

Theoretical foundations

Odontogenesis and Developmental Biology

Human tooth development is a complex, multistage process that begins during the sixth week of embryonic development and continues through adolescence. Odontogenesis involves sequential and reciprocal interactions between the oral epithelium and the underlying neural crest-derived mesenchyme [9]. This process is orchestrated by a cascade of molecular signaling pathways that regulate critical developmental stages: initiation, morphogenesis, cytodifferentiation, and mineralization [10].

The dental lamina, a band of epithelial tissue that invaginates into the underlying mesenchyme, serves as the primordium for tooth development. During the initiation stage, localized thickenings of the dental lamina form tooth buds at specific positions along the jaw, determined by a combinatorial code of homeobox genes and transcription factors [10,11]. Disruptions at any stage of this tightly regulated process can result in numerical, structural, or positional dental anomalies.

The molecular basis of tooth number determination involves a delicate balance between activating and inhibitory signals. Multiple signaling pathways, including bone morphogenetic protein, fibroblast growth factor, sonic hedgehog, and Wingless-related integration site, play crucial roles in dental lamina proliferation and tooth bud initiation [11]. Inhibitory molecules modulate the extent of dental lamina activity, preventing excessive tooth formation [10]. Perturbations in this regulatory balance can lead to either hypodontia (insufficient activation or excessive inhibition) or hyperdontia (excessive activation or insufficient inhibition).

Classification of Numerical Dental Anomalies

Numerical dental anomalies are classified based on the deviation from the normal dental formula. Hypodontia refers to the developmental absence of one to five permanent teeth (excluding third molars), oligodontia involves the absence of six or more teeth, and anodontia represents the complete absence of all teeth [1]. Hyperdontia, conversely, is characterized by the presence of supernumerary teeth beyond the normal complement. Supernumerary teeth are further classified by morphology (conical, tuberculate, supplemental, or odontoma-like), position (mesiodens, paramolar, distomolar), and orientation (vertical, inverted, horizontal) [6].

CHH represents a unique subset where both hypodontia and hyperdontia coexist in the same individual. This paradoxical condition challenges traditional etiological models and suggests complex, possibly multifactorial pathogenic mechanisms [2-4]. The simultaneous occurrence of these opposing anomalies has been documented in both syndromic and non-syndromic presentations, with the latter being more commonly reported in the literature [11].

For the purposes of this review, CHH is defined as the radiographically confirmed co-occurrence of congenital tooth agenesis and supernumerary tooth formation in the same individual, excluding third molars from both anomaly counts. This exclusion of third molars is applied because their absence or excess is governed by distinct evolutionary and developmental mechanisms and is not considered part of the CHH phenotype [1,4]. True congenital absence must be distinguished from delayed eruption - in which a fully formed tooth germ exists but has not yet emerged - and from teeth lost to prior extraction; wherever possible, diagnosis should be based on radiographic evidence of absent or supernumerary tooth germs rather than clinical observation alone. Cases in which teeth were extracted prior to radiographic assessment introduce diagnostic uncertainty and should be clearly identified in study reporting [1,4].

Etiology and pathogenesis

Genetic and Molecular Mechanisms

The exact etiopathogenesis of CHH remains a subject of ongoing academic debate, though accumulating evidence points to a dual disruption of the dental lamina involving both hyperactivity and localized failure [3,9]. Genetic factors play a foundational role, with mutations in several key signaling genes frequently implicated in phenotypic combinations of missing and extra teeth.

Key genes implicated in hypodontia include PAX9, MSX1, WNT10A, and components of the Ectodysplasin (EDA) pathway. PAX9 and MSX1 encode transcription factors critical for early tooth initiation; loss-of-function variants in these genes are associated with posterior tooth agenesis [3]. WNT10A mutations are among the most common genetic causes of non-syndromic hypodontia, particularly affecting anterior and premolar regions [9]. Conversely, hyperactivation of the WNT/β-catenin pathway has been implicated in supernumerary tooth formation, as this pathway promotes dental lamina proliferation and ectopic tooth bud initiation [10,11]. The simultaneous occurrence of loss-of-function and gain-of-function perturbations within the same individual - possibly through complex epistatic interactions or somatic mosaicism - may underlie the dual phenotype observed in CHH. It should be noted that the genetic associations described above are largely extrapolated from studies of isolated hypodontia or isolated hyperdontia; direct evidence linking these gene variants specifically to the co-occurrence phenotype of CHH is limited to case reports and small series. Caution is therefore warranted when attributing the dual phenotype to any single molecular mechanism.

Proposed Dual Disruption Hypothesis

The etiology of CHH remains incompletely understood, and no single mechanism has been definitively established. The proposed dual disruption hypothesis suggests a dual disruption of the dental lamina: localized hyperactivity results in the formation of supernumerary teeth, while simultaneous fragmentation or failure of the lamina leads to the agenesis of permanent teeth [3,9]. This may result from disturbances in the migration, proliferation, and differentiation of neural crest cells and interactions between epithelial and mesenchymal cells during the initiation of odontogenesis [2,9]. The genetic basis for this dual disruption likely involves complex epistatic interactions between multiple genes and environmental factors [3].

Environmental and Syndromic Associations

While genetic factors are paramount, environmental influences during critical developmental windows may contribute to CHH. Proposed environmental factors include maternal infections, nutritional deficiencies, exposure to teratogens, and trauma during pregnancy, though direct causal relationships remain difficult to establish [12]. Additionally, systemic conditions such as subclinical hypothyroidism have been reported in association with CHH, suggesting that endocrine disturbances may play a role in its etiology [13].

CHH can occur as an isolated non-syndromic condition or as part of various genetic syndromes. Syndromic associations include Down syndrome, Ellis-van Creveld syndrome, and cleidocranial dysplasia [3,12]. The occurrence of CHH in identical twins provides compelling evidence for a strong genetic component, though the incomplete penetrance observed in some families suggests multifactorial inheritance involving both genetic and environmental factors [12,14].

Epidemiology

Global Prevalence and Distribution

Concomitant hypo-hyperdontia is an extremely rare condition, with global prevalence estimates ranging from 0.002% to 3.1% across different populations [4,7]. This wide range reflects variations in study methodologies, population characteristics, and diagnostic criteria; specialist referral cohorts tend to yield higher prevalence estimates than population-based radiographic screening studies, and studies differ in whether primary dentition anomalies are included [1,4]. A comprehensive critical appraisal of published data by Chen et al. identified 103 individuals with CHH reported in the English literature, with an average age of 11.29 years at diagnosis [1]. The condition demonstrates a significant male predominance, with men being more frequently affected than women (P < 0.05) [1,8].

In a large retrospective study of 41,648 pediatric patients in China, Qiu et al. reported a CHH prevalence of 0.35% (145 cases), with male patients (102 cases) significantly more affected than female patients (43 cases) (P < 0.001) [8]. Similarly, a Turkish population study of 10,000 individuals found hypodontia prevalence of 3.72%, hyperdontia prevalence of 0.77%, and CHH prevalence of 0.04% [5]. A study of 7,011 children aged 7-12 years in Turkey reported an overall dental number anomaly frequency of 5.14%, with hypodontia at 4.75%, hyperdontia at 0.4%, and CHH observed in only one patient (Table 1) [15].

**Table 1:** Summary of Key Epidemiological Studies on Concomitant Hypodontia and Hyperdontia (CHH) N/A: Not applicable; NR: Not Reported. Source: Data extraction table. Compiled from Chen & Mallineni [1], Qiu et al. [8], Bilgili & Coşkun [5], Kurt & Kara [15], Eshgian et al. [4], Varela et al. [3], Anthonappa et al. [7], and Alshammari et al. [16]. Calculated Prevalence = CHH cases (n) ÷ total sample (N) × 100. ‡ Bilgili & Coşkun [5]: Research Square preprint [Preprint; not peer-reviewed] — data should be interpreted with caution. Note: Chen & Mallineni [1] (published 2017) performed a critical appraisal of cases reported since 1967; inclusion of cases across several decades introduces diagnostic heterogeneity.

Study (Year)	Country	Source Population	Age Range	Sample Size (N)	Study Design	Diagnostic Criteria	Third Molar Excl.	CHH Cases (n)	Reported Prevalence	Calculated Prevalence (n/N × 100)	95% CI	M:F Ratio
Chen & Mallineni (2017) [1]	Multi-national	Literature-derived CHH cases (cases since 1967)	Variable	103 CHH cases (literature)	Critical appraisal	Published case criteria (variable)	NR	103	N/A (case review)	N/A	NR	~2:1
Qiu et al. (2023) [8]	China	Pediatric hospital patients	Not specified	41,648	Retrospective	Clinical + radiographic	NR	145	0.35%	0.35% (145/41,648)	NR	2.4:1
Bilgili & Coşkun (2025) [5]‡	Turkey	Dental hospital patients	Adults + children	10,000	Cross-sectional	Radiographic	NR	4	0.04%	0.04% (4/10,000)	NR	~2:1
Kurt & Kara (2022) [15]	Turkey	Children at dental faculty	7–12 years	7,011	Retrospective	Clinical + radiographic	NR	1	<0.02%	0.014% (1/7,011)	NR	N/A
Eshgian et al. (2021) [4]	USA	Referral/clinic population	Not specified	Not specified	Retrospective	Radiographic	NR	NR	0.002%–0.05%	N/A	NR	Male predominant
Varela et al. (2009) [3]	Spain	Orthodontic patients	Not specified	Not specified	Retrospective	Clinical + radiographic	NR	NR	0.78%	N/A	NR	~1.5:1
Anthonappa et al. (2008) [7]	Multi-national	Literature review + 7 new cases	Not specified	Literature + 7 new cases	Case series & review	Various (literature-derived)	NR	7 (new cases)	0.002%–3.1% (range)	N/A	NR	Male predominant
Alshammari et al. (2025) [16]	Saudi Arabia	Regional cohort (Hail Province)	Mixed	Regional cohort	Cross-sectional	Clinical + radiographic	NR	NR	Not reported separately for CHH	N/A	NR	Not reported

Distribution Patterns

Analysis of tooth distribution in CHH reveals that hypodontia is more common than hyperdontia within affected individuals, with 63% of anomalies representing missing teeth and 37% representing supernumerary teeth (P < 0.05) [1]. The anterior region of both arches is involved in 57% of cases, reflecting the clinical significance of aesthetic zone involvement [1]. Second premolars are the most commonly missing teeth in both maxillary and mandibular arches, while maxillary mesiodens (50%) are the most frequent supernumerary teeth in CHH patients [1,8]. Notably, posterior CHH (involving only posterior teeth) has not been reported in the literature [1].

Arch-Specific Patterns

Congenital permanent tooth loss predominantly involves 1-2 teeth, mainly mandibular lateral incisors and second premolars, with a higher incidence in the mandible than the maxilla (P < 0.001) [8]. Supernumerary teeth are mostly 1-2 in number, located in the maxillary anterior area, conical in shape, with vertical inversion and orthotopic growth patterns [8].

Saudi Arabian and Regional Data

Saudi-specific epidemiological data on CHH are currently sparse. Studies on dental anomalies in Saudi regional cohorts, such as Hail Province, have documented rates of hypodontia and hyperdontia in isolation [8], but no large-scale population-based study of CHH prevalence in Saudi Arabia has been published to date. It has been hypothesised that consanguineous marriage patterns prevalent in Saudi Arabia may theoretically increase the expression of autosomal recessive dental anomalies; however, no published studies have directly linked consanguinity to CHH specifically in Saudi Arabia, and this association remains speculative. Dedicated epidemiological research is therefore warranted. Extrapolation from global or regional data to the Saudi context should therefore be made with caution, and this represents a significant evidence gap.

Clinical presentation and diagnosis

Clinical Features

The clinical presentation of CHH varies depending on the specific teeth involved, the number and type of supernumerary teeth, and the stage of dental development at diagnosis (Figure 1). Common clinical features include asymmetric eruption patterns, prolonged retention of deciduous teeth, unexplained spacing or crowding in the dental arches, and aesthetic concerns related to missing or malformed anterior teeth [6,16]. In the maxillary anterior region, the combination of missing lateral incisors and mesiodens can result in midline diastema, delayed or ectopic eruption of permanent incisors, and compromised smile aesthetics [5,11].

**Figure 1:**
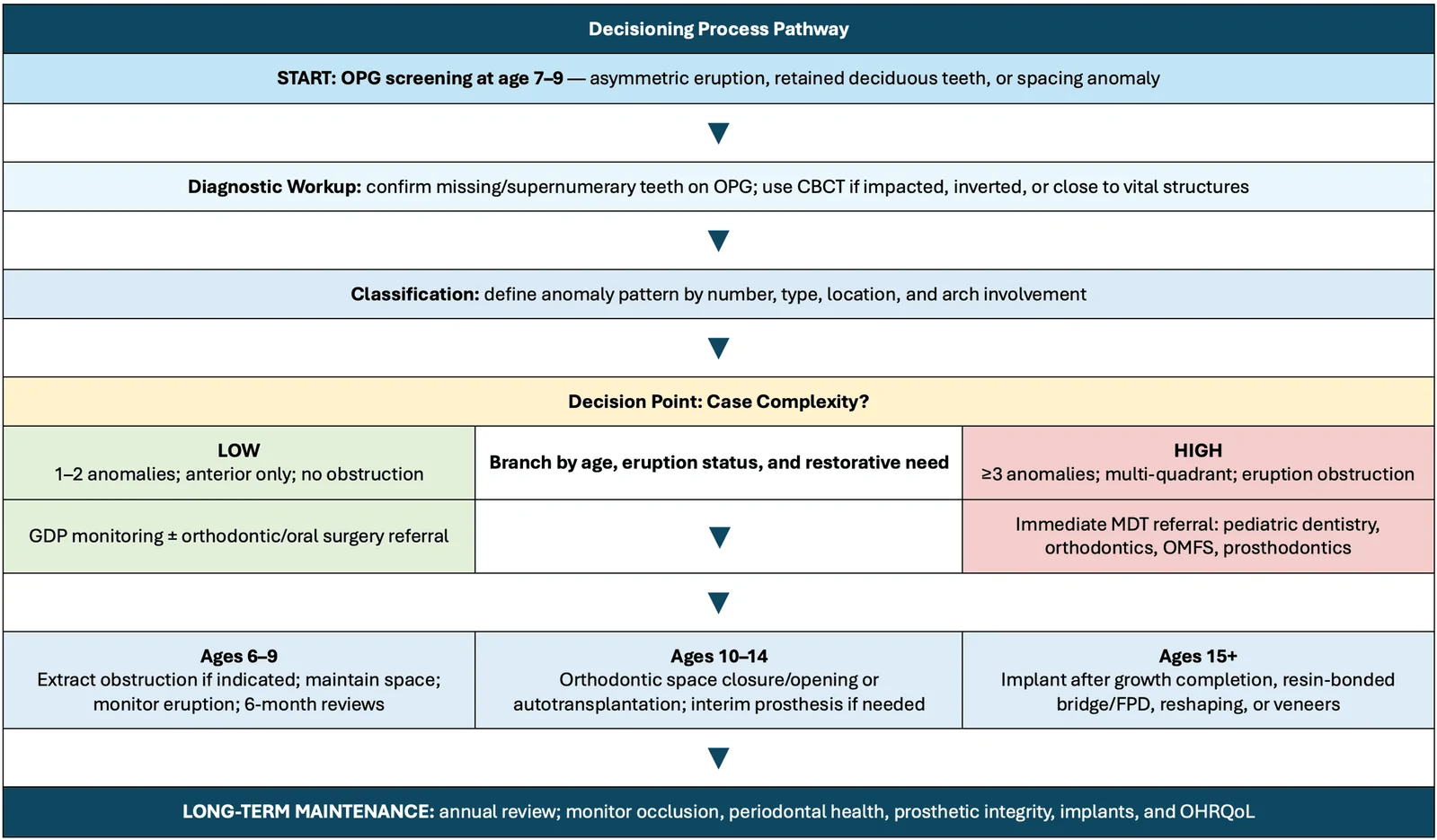
Proposed Clinical Decision-Making Flowchart for Concomitant Hypodontia and Hyperdontia (CHH) OPG: Orthopantomogram, panoramic dental imaging radiograph; CBCT: Cone-Beam Computed Tomography, a specialized 3D medical and dental imaging technique; GDP: General Dental Practitioner; MDT: Multidisciplinary Team; OMFS: Oral Maxillofacial Surgery; OHRQoL: Oral Health-Related Quality of Life

Patients may present with functional complaints such as difficulty in mastication, speech articulation problems, or temporomandibular joint dysfunction secondary to malocclusion [6]. Psychosocial impacts are particularly significant in adolescents, with anterior dental anomalies leading to reduced social confidence, psychological distress, and impaired quality of life [14]. The aesthetic and functional consequences of CHH emphasize the importance of early detection and timely intervention.

Radiographic Assessment

Comprehensive radiographic examination is essential for the diagnosis and management planning of CHH. The mixed dentition phase (ages 6-12 years) represents the critical window for intervention, with routine radiographic screening optimally targeted at ages 7-9 years [8,17], as permanent teeth are actively erupting during this period and interceptive measures yield the most favorable outcomes before eruption pathways are irreversibly compromised [15,16]. Panoramic radiographs (orthopantomograms, OPG) are the primary diagnostic tool, providing a comprehensive view of the entire dentition and allowing visualization of unerupted teeth, supernumerary elements, and developmental anomalies [8,11,15].

In cases where panoramic radiographs reveal potential CHH, supplementary imaging may be necessary. Periapical and occlusal radiographs provide detailed information about specific regions, while cone-beam computed tomography (CBCT) offers three-dimensional visualization of tooth position, orientation, and relationship to adjacent structures [2,17]. CBCT is particularly valuable for surgical planning, allowing precise localization of impacted or inverted supernumerary teeth and assessment of their proximity to vital structures such as the nasal cavity and maxillary sinus [2]. In line with the ALARA (As Low As Reasonably Achievable) principle, CBCT should be reserved for cases where three-dimensional information is expected to directly change clinical management and should not be used as a routine imaging modality [2,17].

For children under 10 years of age with a suspected or confirmed diagnosis, follow-up radiographic examinations at 2-4-year intervals are recommended [3]. When interpreting follow-up radiographs, clinicians should carefully distinguish between: first, delayed mineralization, in which a tooth germ is present but incompletely calcified and may appear absent on early imaging; second, delayed eruption, in which a fully formed tooth has not yet emerged into the oral cavity; and thirdly, true agenesis, in which no tooth germ can be identified across serial radiographic assessments. This distinction is essential for accurate diagnosis and avoids premature labelling of delayed-developing teeth as genuinely absent. The judicious use of radiographs not only enhances the detection of CHH but also leads to changes in the course of management, enabling early interceptive interventions [7].

Clinical management

Multidisciplinary Approach

Evidence note: No randomized controlled trials or CHH-specific comparative studies exist in the current literature. The clinical recommendations presented in this section are extrapolated from studies of isolated hypodontia, isolated hyperdontia, or related dental anomalies and represent author-proposed guidance based on expert consensus rather than direct CHH evidence. Individual recommendations are labelled as evidence-based (supported by studies of analogous conditions), consensus-based (reflecting broad clinical agreement), or author-proposed (based on clinical reasoning) where applicable.

Successful management of CHH requires a coordinated, multidisciplinary approach involving pediatric dentists, orthodontists, oral and maxillofacial surgeons, prosthodontists, and periodontists [6,8,11,15]. The complexity of CHH cases necessitates comprehensive treatment planning that addresses both the immediate concerns (supernumerary tooth removal, space management) and long-term objectives (functional occlusion, aesthetic restoration, periodontal health) [9,12].

Early diagnosis is essential for proper management, and the establishment of a multidisciplinary team at the time of diagnosis facilitates coordinated care [6,8,12]. Treatment protocols depend on multiple factors including the severity of the condition, timing of diagnosis, patient age, available space in the dental arch, and patient and guardian preferences [9]. No standardized treatment protocols exist for CHH due to its rarity and variability, necessitating individualized treatment planning based on careful clinical and radiographic assessment [12].

Surgical Interventions

Removal of supernumerary teeth is often required to clear the eruption path for permanent teeth and prevent complications such as delayed eruption, ectopic positioning, root resorption of adjacent teeth, and cyst formation [6,9,11]. The timing of surgical extraction is critical and depends on the position, orientation, and developmental stage of the supernumerary tooth, as well as the stage of development of adjacent permanent teeth.

For conical supernumerary teeth in the anterior maxilla, early extraction (typically during the mixed dentition phase) is generally recommended to facilitate spontaneous eruption and alignment of permanent incisors [9]. However, for deeply impacted or inverted supernumerary teeth, a more conservative approach with periodic monitoring may be appropriate until the patient is older and can better tolerate surgical intervention under local anesthesia [16].

In cases where hypodontia results in significant alveolar ridge deficiency, bone grafting procedures may be necessary to augment the ridge prior to implant placement [18]. Late diagnosis of CHH may carry significant economic implications at the individual level, as missing lateral incisors can lead to alveolar ridge atrophy that necessitates complex secondary procedures [18]. No CHH-specific cost-effectiveness data currently exist; this observation is extrapolated from the management literature for isolated hypodontia.

Orthodontic Strategies

Orthodontic management of CHH involves critical decisions regarding space management: space closure (canine substitution) versus space opening for prosthetic replacement [9,11,18]. Each approach has distinct advantages and limitations that must be carefully considered in the context of the individual patient’s occlusion, facial profile, and aesthetic expectations.

Space Closure and Canine Substitution

This approach involves orthodontic movement of the canine into the lateral incisor position, followed by reshaping of the canine to mimic lateral incisor morphology. Advantages include superior periodontal health (natural tooth support), avoidance of prosthetic complications, and elimination of the need for lifelong prosthetic maintenance [9,12]. However, canine substitution requires careful attention to occlusal relationships, particularly canine guidance and anterior coupling, and may necessitate reshaping of multiple teeth to achieve optimal aesthetics [9].

Space Opening for Prosthetic Replacement

This approach maintains or creates space for prosthetic replacement of the missing lateral incisor, typically with a dental implant or resin-bonded bridge. Advantages include preservation of natural tooth positions and potentially superior aesthetics in cases where canine substitution would compromise smile design [18]. However, this approach requires long-term prosthetic maintenance, carries risks of implant complications, and may be more costly over the patient’s lifetime [18].

The choice between these strategies should be based on comprehensive assessment of occlusal relationships, skeletal pattern, soft tissue profile, patient age, and long-term prognosis [9,18]. In cases involving multiple missing teeth, a combination of space closure and prosthetic replacement may be optimal [9].

Restorative and Prosthetic Solutions

Restorative excellence is essential for achieving optimal aesthetic outcomes in CHH management. Transforming canines into lateral incisors via composite build-ups or veneers requires high technical skill to ensure aesthetic symmetry, appropriate tooth proportions, and natural appearance [9,18]. Composite resin restorations offer a conservative, reversible approach for reshaping canines and can be modified or replaced as needed [9,10].

For cases where space opening is chosen, prosthetic options include resin-bonded bridges (Maryland bridges), conventional fixed partial dentures, removable partial dentures, and dental implants [9,10]. Dental implants are generally considered the gold standard for single-tooth replacement in adults, offering superior aesthetics, function, and longevity compared to other prosthetic options [10]. However, implant placement in adolescents must be delayed until skeletal growth is complete - typically age 18-21 for men and 16-18 for women - necessitating interim prosthetic solutions during the growth period (Table 2) [10].

**Table 2:** Comparison of Restorative Options for Anterior Hypodontia in Concomitant Hypodontia and Hyperdontia (CHH) Source: Compiled from the study by Ruschel et al. [9], Bharti et al. [10], Gupta & Popat [11], Mallineni et al. [12], and Anthonappa et al. [7]. Evidence levels based on available case series, cohort studies, and expert consensus; no randomized controlled trials are available for this rare condition.

Restorative Option	Indications	Advantages	Disadvantages	Evidence Level	Long-Term Prognosis
Orthodontic space closure (canine substitution)	Adequate canine morphology; Class I/mild Class II occlusion	No prosthesis required; superior periodontal health; no maintenance burden	Requires canine reshaping; occlusal adjustment; not suitable for all cases	Moderate (case series, cohort studies)	Good with appropriate reshaping
Dental implant	Completed skeletal growth (≥18 yrs); adequate bone volume	Gold standard aesthetics; longevity; independent of adjacent teeth	Delayed until growth complete; surgical risk; cost; requires bone volume	Moderate (prospective studies)	Excellent in adults with adequate bone
Resin-bonded bridge (Maryland bridge)	Adolescents awaiting implant; minimal tooth preparation preferred	Conservative; reversible; good interim solution	Risk of debonding; limited longevity; aesthetic limitations	Low-moderate (case series)	Moderate; suitable as interim
Conventional fixed partial denture	Multiple missing teeth; adjacent teeth require restoration	Established technique; durable	Requires preparation of adjacent teeth; irreversible	Moderate	Good with maintenance
Removable partial denture	Growing patients; multiple missing teeth; interim solution	Non-invasive; adjustable	Poor patient acceptance in young patients; maintenance required	Low (expert consensus)	Fair; transitional use only
Composite resin build-up/veneer	Canine substitution cases; minor morphology correction	Conservative; reversible; low cost	Wear over time; periodic replacement needed	Low-moderate (case series)	Moderate with regular maintenance

Preventive measures, including fissure sealants for permanent molars and preventive resin restorations, should be implemented to maintain oral health during the extended treatment period often required for CHH management [11,16].

Clinical Decision-Making and Management Algorithms

The development of evidence-based decision algorithms for CHH management is challenging due to the rarity of the condition and the lack of large-scale comparative studies. However, general principles can guide clinical decision-making. Early diagnosis during the mixed dentition phase remains the cornerstone of management, with routine panoramic radiography for children aged 7-9 years enabling timely identification of CHH and initiation of treatment planning [15,16]. Where supernumerary teeth obstruct eruption paths or cause complications, early extraction is indicated, while asymptomatic, deeply impacted supernumeraries may be managed conservatively with regular monitoring [9,11]. Once the supernumerary component has been addressed, a comprehensive orthodontic assessment should be undertaken to determine the optimal space management strategy - either space closure or space opening - based on the patient’s occlusion, skeletal pattern, and preferences [9,18]. Definitive treatment, whether space closure or prosthetic replacement, is implemented during adolescence, with final restorative work completed following orthodontic alignment [9,10]. Long-term follow-up remains essential throughout, with regular review to monitor treatment stability, periodontal health, and prosthetic integrity, and lifelong maintenance for implant-supported restorations [10].

Public health considerations in Saudi Arabia

The Saudi Arabian healthcare context is relevant as a case example of integrating rare dental anomaly management into a transforming primary care system. Under the Health Sector Transformation Program of Vision 2030, the Saudi dental model is evolving toward a Value-Based Healthcare (VBHC) framework that prioritizes prevention and early intervention [18]. Within this context, the GDP’s role as a diagnostic gatekeeper for developmental dental anomalies is of practical importance. The Vision 2030 transformation includes several initiatives directly relevant to CHH management. Primary, secondary, and tertiary care are being integrated within unified geographic healthcare clusters to facilitate seamless patient referrals and coordinated multidisciplinary care [18]. Primary healthcare centers (PHCs) are being enhanced with advanced diagnostic capabilities, including digital radiography, to enable early detection of dental anomalies at the point of first contact [16]. Comprehensive workforce development programs are equipping GDPs with enhanced diagnostic skills in pediatric dentistry and developmental anomalies [16,18]. The implementation of electronic health records and telemedicine platforms further supports specialist consultation and treatment coordination across care settings [18]. Underpinning these efforts, performance indicators for early detection of developmental anomalies and timely referral to specialist care are being established to ensure accountability and drive quality improvement [18].

From a primary care perspective, standardized screening protocols, including routine panoramic radiography for children aged 7-9 years, would enhance early detection of CHH and other dental anomalies in Saudi PHCs [15,16]. Clear referral pathways between primary care GDPs and specialist orthodontic and surgical services are essential for timely and coordinated care. A potential economic benefit of early detection has been proposed: timely diagnosis may reduce the need for complex secondary procedures such as bone grafting prior to implant placement [18]. However, CHH-specific cost-effectiveness evidence is currently lacking, and this rationale should be understood as extrapolated from isolated hypodontia management literature rather than directly demonstrated for CHH.

It should be noted, however, that CHH is a rare condition (global prevalence 0.002%-3.1%), and its direct impact on national healthcare burden is proportionally limited. The individual-level clinical and psychosocial burden of CHH, which can be substantial for affected patients, should not be conflated with population-level public health significance, which must remain proportionate to the condition’s rarity and the current absence of large-scale epidemiological data. Its relevance to public health policy lies primarily in its value as a model for rare developmental anomaly management and as an indicator of primary care diagnostic capacity, rather than as a major population-level health priority.

Clinical implications

The management of concomitant hypo-hyperdontia represents a sophisticated intersection of biological rarity and clinical necessity. The paradoxical nature of CHH, simultaneous tooth agenesis and supernumerary tooth formation, challenges traditional etiological models and necessitates a nuanced understanding of developmental biology and genetic mechanisms. While genetic factors play a foundational role in tooth number anomalies, the precise mechanisms underlying the dual disruption of the dental lamina in CHH remain incompletely understood [3,10].

The clinical management of CHH requires individualized treatment planning based on comprehensive assessment of multiple factors, including the specific teeth involved, the patient's age and developmental stage, occlusal relationships, aesthetic expectations, and long-term prognosis. The lack of standardized treatment protocols reflects both the rarity of the condition and the heterogeneity of clinical presentations [13]. However, general principles, early diagnosis, multidisciplinary collaboration, evidence-based decision-making, and patient-centered care, provide a framework for optimal management.

The choice between space closure and space opening strategies for missing lateral incisors remains a subject of clinical debate, with both approaches offering distinct advantages and limitations [7]. Recent evidence suggests that space closure with canine substitution may offer superior long-term periodontal outcomes and reduced prosthetic complications, though aesthetic considerations may favor space opening in selected cases [7]. Long-term comparative studies are needed to establish evidence-based guidelines for treatment selection.

Public health considerations

From a public health perspective, CHH serves as an important indicator of healthcare system capacity and effectiveness. The ability to detect rare developmental anomalies at the primary care level, coordinate multidisciplinary specialist care, and achieve optimal long-term outcomes reflects the maturity and sophistication of the healthcare infrastructure. In the context of Saudi Arabia's Vision 2030 healthcare transformation, the management of CHH exemplifies the shift from reactive, hospital-based care to proactive, integrated, value-based care [7].

The economic implications of CHH management extend beyond individual patient costs to encompass broader healthcare system efficiency and resource allocation. Early detection and interceptive treatment represent high-value interventions that prevent costly downstream complications and improve long-term outcomes [7]. The implementation of systematic screening programs, clear referral pathways, and evidence-based treatment protocols can optimize resource utilization and reduce the overall economic burden of CHH on the healthcare system.

The psychosocial dimensions of CHH highlight the importance of a holistic, patient-centered approach to care. Dental anomalies affecting the aesthetic zone have profound impacts on quality of life, particularly during adolescence, and successful management must address both functional and psychosocial outcomes [9]. The integration of psychological support and patient education into CHH management protocols can enhance treatment acceptance, compliance, and overall satisfaction.

Research recommendations

Several important research gaps remain in our understanding of CHH. First, large-scale epidemiological studies are needed to establish accurate prevalence estimates for different populations, including the Saudi Arabian population. The establishment of national dental registries and systematic data collection through Vision 2030 initiatives provides an opportunity to generate robust epidemiological data on rare conditions like CHH.

Second, genetic and molecular research is needed to elucidate the precise mechanisms underlying the dual disruption of the dental lamina in CHH. Whole-exome sequencing and genome-wide association studies in affected individuals and families may identify novel genetic variants and pathways involved in CHH pathogenesis. Understanding these mechanisms could lead to improved genetic counseling, risk prediction, and potentially preventive interventions.

Third, comparative effectiveness research is needed to establish evidence-based guidelines for treatment selection in CHH. Long-term prospective studies comparing space closure versus space opening strategies, different prosthetic options, and various treatment timing protocols would provide valuable evidence to guide clinical decision-making. Patient-reported outcomes, including quality of life and satisfaction measures, should be incorporated into these studies to capture the full spectrum of treatment impacts.

Fourth, implementation research is needed to optimize the integration of CHH screening and management into primary care settings. Studies evaluating different screening protocols, referral pathways, and care coordination models can inform the design of effective and efficient healthcare delivery systems for rare developmental anomalies.

Finally, health economics research is needed to evaluate the cost-effectiveness of different CHH management strategies and screening programs. Such analyses can inform resource allocation decisions and support the business case for investing in early detection and prevention programs within the Vision 2030 framework.

Limitations

This review has several important limitations that should be considered when interpreting its findings.

Methodological Limitations

This is a narrative review without systematic search methodology, formal quality assessment, or risk-of-bias evaluation. The literature selection was not exhaustive and may be subject to selection bias. The evidence synthesis is interpretive, and conclusions represent the authors’ assessment of the available literature rather than a formally weighted analysis.

Evidence Base Limitations

CHH is an extremely rare condition, and the majority of available evidence derives from case reports and small case series rather than controlled studies. No randomized controlled trials comparing management approaches exist. Significant heterogeneity in diagnostic criteria, classification systems, and reporting standards across studies limits comparability and generalizability of prevalence estimates.

Geographic and Population-Specific Limitations

Saudi-specific epidemiological data on CHH are currently absent from the published literature. Prevalence estimates and clinical characteristics reported in this review are derived predominantly from Asian, European, and North American populations and may not accurately reflect the Saudi or Middle Eastern context. Genetic, environmental, and healthcare access factors may differ substantially across populations.

Clinical Algorithm Limitations

The proposed clinical decision-making framework and management pathways are based on expert consensus and extrapolation from related conditions rather than validated, CHH-specific protocols. No prospective validation of these frameworks has been performed, and individual patient factors may necessitate deviation from the suggested pathways.

Healthcare System Analysis Limitations

The discussion of the Saudi Arabian healthcare context is based on policy documents and early implementation reports rather than outcome data. No empirical evaluation of the impact of Vision 2030 healthcare changes on CHH detection or management outcomes is available.

Psychosocial Impact Limitations

Quantitative data on psychosocial outcomes specific to CHH are limited; most evidence is extrapolated from studies of isolated hypodontia or other dental anomalies. No validated, CHH-specific quality-of-life instruments currently exist.

## Conclusions

CHH is a rare numerical dental anomaly defined by the radiographically confirmed co-occurrence of congenital tooth agenesis and supernumerary tooth formation in the same individual, with a consistent male predominance and a predilection for the anterior aesthetic zone. Its proposed molecular basis, the dual disruption hypothesis involving PAX9, MSX1, WNT10A, and related WNT signaling, remains incompletely understood, and the available genetic evidence is largely extrapolated from studies of isolated hypodontia or hyperdontia rather than CHH-specific investigations.

Multidisciplinary management, initiated through early diagnosis during the mixed dentition phase, offers the most favorable clinical outcomes. The decision-making framework presented in this review is author-proposed and based on expert consensus and extrapolation from analogous conditions; prospective validation is required before it is adopted as an evidence-based standard. The public health relevance of CHH lies primarily in its value as a model for rare developmental anomaly management and as an indicator of primary care diagnostic capacity. Future research priorities include Saudi-specific epidemiological studies, CHH-targeted molecular genetic investigation, prospective comparative effectiveness trials, and CHH-specific cost-effectiveness analyses.
